# Barriers and facilitators for the implementation of medication safety recommendations: focus groups with stakeholders

**DOI:** 10.1007/s11096-026-02141-z

**Published:** 2026-04-30

**Authors:** Mirthe A. M. Oude Lansink, Marcia Vervloet, Lise van Tholen, Marloes Dankers, Mette Heringa, Bart J. F. van den Bemt, Liset van Dijk, Victor J. B. Huiskes

**Affiliations:** 1 Department of Pharmacy, Sint Maartenskliniek, Nijmegen, the Netherlands; 2 Department of Research, Sint Maartenskliniek, Nijmegen, the Netherlands; 3 Department of Pharmacy, Radboudumc, Nijmegen, the Netherlands; 4 Netherlands Institute for Health Services Research, Utrecht, the Netherlands; 5 Faculty of Science and Engineering, Department of Pharmaco Therapy, Groningen Research Institute of Pharmacy, Epidemiology & Economics (PTEE), University of Groningen, Groningen, the Netherlands; 6 Dutch Institute for Rational Use of Medicine, Utrecht, the Netherlands; 7 SIR Institute for Pharmacy Practice and Policy, Leiden, the Netherlands

**Keywords:** Drug-related side effects and adverse reactions, Hospitalisation, Implementation science, Medication errors, Patient readmission, Quality of health care

## Abstract

**Introduction:**

Multiple medication safety recommendations to reduce preventable medication-related hospitalisations have been introduced in the Netherlands. Still, these hospitalisations remain prevalent. This might be due to suboptimal implementation in clinical practice. Therefore, more insight is needed into barriers and facilitators for the implementation of these medication safety recommendations across healthcare sectors.

**Aim:**

This study aimed to identify barriers and facilitators for implementing medication safety recommendations across healthcare sectors from the perspective of various stakeholders.

**Method:**

A selection of 17 Dutch medication safety recommendations targeting medications responsible for a large proportion of medication-related hospitalisations was evaluated in focus groups to identify barriers and facilitators to their implementation. These included recommendations for: reducing fall risk in elderly; initiating prophylactic medication; monitoring patients at risk of electrolyte disorders; clarifying responsibilities for patient care; informing patients about alarm symptoms; and prescribing antithrombotics on strict indication. Stakeholders were selected through purposive sampling based on their profession and healthcare sector. Transcripts of audio-recordings were analysed inductively, after which implementation factors were categorised using the Consolidated Framework for Implementation Research.

**Results:**

Thirty stakeholders were divided into five focus groups. These included community pharmacists (n = 9), general practitioners (n = 4), hospital pharmacists (n = 3), representatives from healthcare knowledge organisations (n = 3), patient representatives (n = 2), medical specialists (n = 2), nurses (n = 2), and other stakeholders (n = 5). Forty-nine barriers and facilitators were identified. Key themes were lack of specified responsibilities, limited information exchange, local collaboration and protocol adherence. Recommendations with a lower level of implementation often required collaboration with multiple healthcare providers, whereas recommendations with a higher level of implementation were frequently supported by clinical decision support systems.

**Conclusion:**

A broad range of barriers and facilitators to the implementation of medication safety recommendations was identified. Overall, the findings highlight the need for recommendations with specified responsibilities, improved information exchange, strengthened local collaboration, and increased protocol adherence.

**Supplementary Information:**

The online version contains supplementary material available at 10.1007/s11096-026-02141-z.

## Impact statements

- This study is the first to explore views of stakeholders from multiple disciplines and professions on a wide range of barriers and facilitators affecting the implementation of medication safety recommendations. The findings support policymakers and healthcare providers to identify which barriers to address and which facilitators to leverage to improve the implementation of medication safety recommendations, ultimately contributing to improved patient outcomes.
- Improving the implementation of medication safety recommendations requires recommendations that specify which healthcare provider is responsible for each action involved in evaluating and monitoring a patient’s treatment. Furthermore, data exchange between information systems should be optimised, healthcare providers should strengthen their local collaboration, and protocol adherence must be increased.

## Introduction

Medication-related hospital readmissions are common, causing physical and emotional stress for patients, increasing healthcare providers’ (HCPs) workload and burdening the healthcare system financially [1, 2]. Adverse drug reactions (ADRs) and other medication-related problems (MRPs) account for around 10–20% of unplanned hospitalisations among older adults [3]. Furthermore, a systematic review reported that 21% (range 3–64%) of hospital readmissions are medication-related, and 69% (range 5–87%) of these were considered preventable. Examples of preventable cases include ADRs that could have been avoided through prophylactic co-medication or adequate monitoring [3].

To improve medication safety in the Netherlands and prevent unnecessary harm to patients, a series of studies was conducted, resulting in policy-level and clinical recommendations [4–6]. The clinical recommendations outline actions HCPs should perform to optimise medication safety and include: decreasing fall risk in the elderly; initiating prophylactic medication (e.g., adding a laxative alongside opioid therapy); monitoring patients at risk of electrolyte disorders; clarifying responsibilities regarding a patient’s treatment; informing patients about alarm symptoms; and prescribing antithrombotic treatment on strict indication. These recommendations were formulated after identification of major ADRs causing hospitalisations and analysis of risk factors and risk-reduction strategies [7]. This first analysis was published in 2008 [5]. A follow-up study showed that five years after the introduction of these medication safety recommendations, medication-related hospital admissions were not reduced [4]. Still, 49,000 patients were hospitalised due to medication use in 2013, over half of these admissions were deemed potentially preventable [4]. No further research exists on medication-related hospital readmissions in the Netherlands. Despite emphasis on implementing these recommendations [4, 7], implementation in clinical practice seems incomplete. Hence, more knowledge is required on factors influencing the implementation of medication safety recommendations.

Multiple medication safety initiatives exist addressing elements of the medication safety recommendations, such as medication reviews, prescribing pharmacists, patient counselling interventions (e.g., teach-back), stewardship programmes, machine-learning technologies and quality indicators [8–14]. Although these initiatives potentially improve medication safety, they do not ensure implementation of all medication safety recommendations. Additionally, they often focus on specific patients, phases of medication use, and specific healthcare settings, whereas medication safety recommendations formulated by the Harm-Wrestling workgroup and Sturkenboom et al. require system-wide implementation beyond individual initiatives [4, 5]. Consequently, broader organisational changes are needed to achieve full implementation, in line with the World Health Organization’s *Medication Without Harm* report [15]. Such changes should be guided by knowledge of barriers and facilitators, ideally identified by HCPs and other stakeholders [16].

A well-known barrier to the implementation of medication safety recommendations is inadequate information transfer between HCPs, particularly between secondary and primary care [17, 18]. In contrast, clinical decision support systems (CDSS) have been shown to positively contribute to the implementation of these recommendations [19, 20]. However, limited studies assessed barriers and facilitators to implementing clinical medication safety recommendations among various professionals and across multidisciplinary settings. Insight into these factors supports policymakers and HCPs in identifying which barriers to address and which facilitators to leverage to improve implementation of medication safety recommendations.

### Aim

This study aimed to identify barriers and facilitators affecting the implementation of medication safety recommendations in clinical practice from the perspective of various stakeholders.

## Method

### Study design

A qualitative focus group study was performed between September 2023 and February 2024 to assess barriers and facilitators to the implementation of selected medication safety recommendations. A preparatory phase preceded the focus groups to select and classify the recommendations on their level of implementation, which informed the focus group discussions. This study is reported according to the Consolidated Criteria for Reporting Qualitative Research (COREQ) [21].

### Preparation phase

#### Selection of medication safety recommendations

Clinical medication safety recommendations were selected from the medication safety recommendations formulated in the Harm-Wrestling report and the follow-up report by Sturkenboom et al. [4, 5]. Policy and preconditional recommendations were excluded because these did not concern clinical actions. After exclusion of non-clinical recommendations, 33 clinical medication safety recommendations remained. A further selection was made, as assessing all recommendations was not feasible. This selection was based on the expected impact of each recommendation on medication safety, taking into account the size of the affected population, the potential harm for affected patients and an initial estimate of the current implementation status. To capture insights from both well- and poorly implemented recommendations, both types were included. Two senior researchers (BvdB, MH), both pharmacists, independently conducted the assessment of population size, potential harm for patients and implementation status, after which they compared and discussed any discrepancies. A third senior researcher (VH), also a pharmacist, reviewed the selection, after which consensus was established. Ultimately, 17 unique recommendations were selected (Table 1).

**Table 1:** Selected medication safety recommendations

Medication safety recommendation		Types of recommended medication safety actions							Level of implementation
Theme^a^	Description	Selection patient at risk^b^	Patient counselling	Communication between HCPs	Laboratory test	Medication start/stop/ change	Responsibility agreements	Periodic assessment	
Antithrombotic therapy	Pharmacy informs anticoagulant service about initiation and discontinuation of medication interacting with coumarin [5]	x		x					High
	Antithrombotic medication, and combinations of, are prescribed only on strict indication. The indication and therapy duration of antithrombotic and other risk medication are communicated by the prescriber to other involved HCPs [4, 5]	x		x		x			Low
Initiating and stopping prophylactic medication for risk patients	Adding PPI to patients using NSAID/acetylsalicylic acid at risk of gastrointestinal bleeding. Patients are informed about alarm symptoms [5]	x	x			x			High
	Informing patients about the importance of therapy adherence to PPI. This PPI is discontinued along with the NSAID/acetyl salicylic acid [5]	x	x			x			High
	Osteoporosis prophylaxis is initiated when indicated due to corticosteroid use. This prophylaxis is discontinued along with the corticosteroid if no longer indicated [5]	x				x			Moderate
	Adding laxatives to opioid therapy, and evaluation whether the current laxative is sufficient to prevent constipation [5]	x				x			High
Contraindications	No NSAID in case of contraindications (i.e., cardiovascular disease, kidney insufficiency, decreased effective circulating volume, use of RAS-inhibitors and/or diuretics). If there is no alternative, NSAID is prescribed for short-term use with kidney function monitoring. Patients are informed about alarm symptoms [5]	x	x		x	x			Moderate
	Glibenclamide is not to be prescribed to patients ≥ 75 years old due to hypoglycaemic risk [5]	x				x			High
Monitoring patients at risk	Monitoring of sodium/potassium levels and kidney function after initiation of a diuretic or a RAS-inhibitor in patients at risk for electrolyte disorder or decreased kidney function [5]	x			x				Low
	Monitoring patients at increased risk due to their medication for electrolyte disorders, kidney insufficiency, syncope or hypotension, especially in case of vomiting or diarrhoea. Laboratory tests must be collected, documented and shared with involved HCPs [4]	x		x	x				Low
Informing patients about alarm symptoms	Informing patients at risk of an electrolyte disorder about alarm symptoms and risk situations (e.g., infection, diarrhoea) [5]	x	x						Low
	Informing patients using an anticoagulant about the risks of intercurrent diseases or changes in lifestyle/nutrition, and alarm symptoms of a gastrointestinal bleeding [5]	x	x						Low
	Users of oral blood glucose-lowering sulfonylurea derivates are informed about the risks of unusual physical exertion and nutrition changes, and how to manage these factors. Patients are informed about alarm symptoms in case of hypoglycaemic risks [5]	x	x						Low
Fall prevention	Psychotropic drugs are only initiated in elderly when there is a strict indication. Fall risk in elderly is assessed by the GP [5]	x				x		x	Low
	Psychotropic drugs and fall-risk increasing cardiovascular medication are not continued unnecessarily. The prescriber evaluates this periodically after initiation. Psychotropic and cardiovascular drugs are evaluated yearly in case of long-term use [5]	x				x		x	Low
	Chronic users of benzodiazepines or related substances are encouraged to discontinue or reduce the dosage by terms of a discontinuation letter [5]	x	x			x			Low
Responsibility agreements	Prescribers establish who holds primary responsibility for a patient’s therapy, document this, and communicate this to involved HCPs, including the pharmacist. When initiating high-risk medication not intended for long-term use, the patient, other prescribers and the pharmacist are informed about the intended therapy duration. The responsibility for periodic monitoring and evaluation of medication initiated in secondary care is discussed and documented. Elderly with polypharmacy receive periodic medication reviews [5]		x	x			x	x	Low

GP = general practitioner, HCP = healthcare provider, NSAID = non-steroidal anti-inflammatory drug, PPI = proton-pump inhibitor, RAS = renin-angiotensin system. For each recommendation, the identified medication safety action types and the assessed level of implementation in the Netherlands are shown

^a^Recommendations were sorted by theme to provide a comprehensive overview

^b^Assessing whether the medication safety recommendation applies to the specific patient

#### Recommended medication safety actions

Although the recommendations involve different medications, HCPs and contexts, HCPs often perform similar clinical actions to adhere to the recommendations (e.g., adding prophylactic medication, counselling patients). We therefore identified the types of actions that were required for each recommendation. Identification was performed independently by two senior researchers (VH, MV) and one junior researcher (MOL). Discrepancies were discussed after which consensus was achieved. Seven different types of recommended medication safety actions were identified: selecting patients at risk; patient counselling; communication between HCPs; laboratory tests; medication start/stop/change; responsibility agreements between HCPs; and periodic assessment (Table 1).

#### Level of implementation

The level of implementation (high, moderate, low) of the selected medication safety recommendations was assessed using three methods. First, a desktop research was performed of scientific and grey literature on the level of implementation in the Netherlands (search strategy and in- and exclusion criteria are described in Supplementary File 1). Second, four researchers (VH, BvdB, MH, MOL), all (senior) researchers and pharmacists, assessed the level of implementation based on desktop research results and their knowledge and experience. This resulted in a preliminary classification: seven of the 17 medication safety recommendations were classified as well or moderately implemented (Table 1), while the remaining ten were classified as poorly implemented. Third, at the start of focus group discussions, stakeholders were asked whether they agreed or disagreed with this preliminary classification. For the final classification, stakeholder opinions were weighted more heavily than expert opinions and desktop research findings, depending on the quality of available literature.

### Focus groups

#### Participants

Since the medication safety recommendations are multidisciplinary and multisectoral, we aimed to include 50 stakeholders to ensure representation across a range of professional backgrounds and healthcare sectors. These included HCPs from general practice, community and outpatient pharmacy, specialist care, anticoagulant care, nursing home care, home care, mental health care and disability care. We aimed to include stakeholders from both clinical practice and policymaking roles, as implementation involves multiple levels [16]. Representatives from patient and healthcare knowledge organisations were also invited. Stakeholders were purposively sampled based on these criteria and recruited via email by the research team, consisting of researchers experienced in implementation and/or medication safety. In addition, open online invitations were distributed via a professional social media platform. Participants did not receive incentives.

#### Focus groups

Five focus groups were conducted at Nivel, Netherlands Institute for Health Services Research, and moderated by experienced researchers (BvdB, MD, MH, MV and CvdS). Stakeholders were grouped according to the medication safety recommendations discussed, as certain professions and healthcare sectors were particularly relevant to specific recommendations. For example, patient representatives were included in focus groups addressing recommendations related to patient counselling. In the first round, barriers and facilitators for recommendations with moderate or high levels of implementation were discussed. The second round focused on those with a low level of implementation. Discussions were guided by a topic guide developed by MD, MV and MOL (Supplementary File 2), which was based on the Consolidated Framework for Implementation Research (CFIR). CFIR categorises factors that influence implementation into six domains: innovation (i.e., the recommendation itself), outer setting (i.e., influences on a national level), inner setting (i.e., within the organisation and its surrounding environment), roles of individuals (i.e., how involved HCPs fulfil their roles), characteristics of individuals (i.e., motivation and capability of involved HCPs), and the implementation process (i.e., activities to support implementation) [22]. CFIR was used to ensure that all implementation domains were addressed during discussions. At the start of each round, stakeholders were asked whether they agreed with the assigned level of implementation for each recommendation. Both rounds lasted 40 min., and each recommendation was discussed in two separate focus groups. Prior to group discussions, stakeholders individually noted potential barriers and facilitators to form their own initial views. All sessions were audio recorded, for which stakeholders provided informed consent after receiving written information about the study. Audio recordings were transcribed verbatim.

#### Outcomes

The main outcome of this study was a set of identified barriers and facilitators to the implementation of medication safety recommendations. Secondary outcomes were a) identified barriers and facilitators for each type of recommended medication safety action and b) the extent to which these factors influenced recommendations with high or low implementation levels, expressed as the percentage of recommendations for which each factor acted as a barrier and/or facilitator. Barriers and facilitators were also mapped to the CFIR domains to examine their distribution across implementation domains, thereby highlighting domains of interest [22].

#### Data analysis

Focus group transcripts were analysed using content analysis in Atlas.ti 9. A junior researcher (MOL) and a Pharmacy Master’s student (LvT) coded the transcripts to identify implementation barriers and facilitators, with ongoing supervision through regular discussions with two senior researchers (VH and MV). Barriers and facilitators were coded inductively by MOL and subsequently mapped to CFIR domains in close consultation with VH and MV. Discrepancies were discussed until consensus was reached. The assessment of which types of recommended actions were influenced by specific barriers or facilitators was performed by MOL based on the contextual information in the transcripts and discussed with VH and MV.

### Ethics approval

Stakeholders provided written informed consent prior to the focus groups, after receiving written information about the study. The study did not fall within the scope of the Medical Research Involving Human Subjects Act as no risk was involved with participation, no patients were involved and no personal or identifiable data from participants were collected [23]. Therefore, no ethical approval was required.

## Results

### Participants

A total of 83 stakeholders from eight healthcare sectors were invited by email to participate in the focus groups, in addition to the open online invitations through which stakeholders could self-register. Thirty stakeholders agreed to participate. Participants represented the following professions: community pharmacists (n = 9), general practitioners (GPs) (n = 4), hospital pharmacists (n = 3), representatives from healthcare knowledge organisations (n = 3), patient representatives (n = 2), medical specialists (n = 2), nurses (n = 2), and other stakeholders (n = 5). Of the twenty HCPs, 14 (70%) were practising HCPs, and six primarily worked in policymaking roles. Stakeholders were divided into five groups, resulting in 5–8 stakeholders per group.

### Distribution of the barriers and facilitators over the CFIR domains

**Fig. 1:**
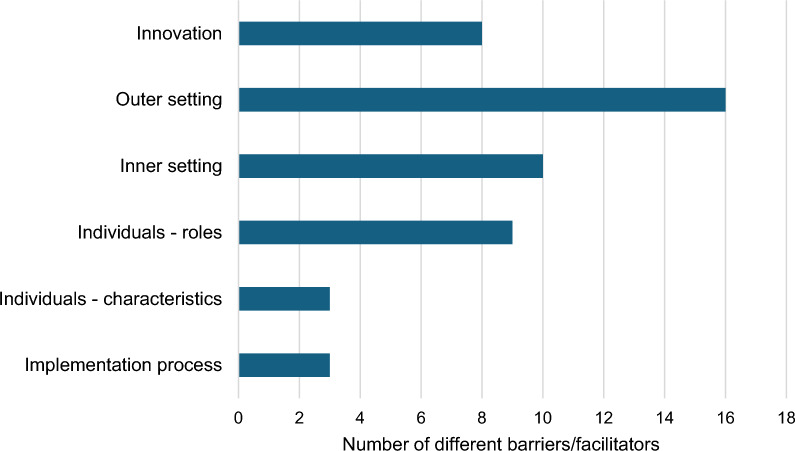
Distribution of the identified barriers and facilitators over the CFIR domains [22]. Innovation = the recommendation itself; outer setting = national influences; inner setting = influences from within the organisation and its surrounding environment; roles of individuals = how involved HCPs fulfil their roles; characteristics of individuals = characteristics of involved HCPs; implementation process = activities aimed at enhancing the implementation

All participants agreed with the assigned level of implementation for the medication safety recommendations. A great variety of implementation factors was identified (N = 49), acting as barriers, facilitators, or both. Outer setting factors accounted for the largest number of barriers and facilitators, as many were related to policy, digital infrastructure and communication at a national level (see Fig. 1).

### Context in which barriers and facilitators influenced implementation for each CFIR domain

#### Innovation

Regarding the recommendations themselves, unclear phrasing and a lack of clearly specified responsibilities were mentioned as barriers. Stakeholders reported uncertainty about what actions to take and by whom. Recommendations were also frequently described as complex, particularly when extensive patient medical data were required to perform the recommended action. These data were not always available or could not be integrated into digital information systems, especially when recommendations required non-protocolled, individualised assessments. In such cases, HCPs need to weigh multiple patient-specific factors, for example, when assessing whether a patient is fit to receive fall-risk increasing medication. In contrast, recommendations that were clearly phrased and required limited evaluation of patient factors were considered facilitators. Stakeholders additionally noted that their adherence to recommendations was lower when they perceived little relative advantage. For example, when they rarely encountered patients at risk or when evidence that following the recommendation would benefit patients’ health was lacking.

*Patient representative: “The patient receives oral and written information. But to me, that’s not entirely concrete. Who is actually going to do that? Will it be the pharmacist or the prescriber? So that they can also monitor themselves to see whether things are going well. I think that could be made a bit more specific.”*

#### Outer setting

In addition to factors related to the recommendation itself, stakeholders indicated that recommendations were difficult to implement when implementation required information from another HCP. They reported limited exchange of medical information, particularly from secondary to primary care, leaving general practices and community pharmacies without essential medical information. Specifically, GPs reported lacking an overview of patients’ medication therapy and noted that discharge information is often delayed and incomplete. Pharmacists reported limited access to recent laboratory results, treatment indications, and contraindications. An underlying barrier reported to have contributed to these issues was the variety of digital healthcare information systems used, which barely communicate with each other. Additionally, prescribers often did not consider it necessary for pharmacists to have access to treatment indications. Privacy regulations and missing information on over-the-counter medication use were also mentioned to have contributed to gaps in knowledge about contraindications and patients’ medication use. Furthermore, pharmacists stated that they were hindered by a lack of reimbursement for pharmaceutical care activities.

*General practitioner: “I don’t know whether a patient has been discharged, what medication has been changed and what agreements have been made. However, the patient considers me their primary clinician again, because they’re back home.’’*

#### Inner setting

Local collaboration across healthcare sectors was identified by stakeholders as a key factor affecting implementation. Lack of collaboration often served as a barrier, whereas effective collaboration acted as a facilitator. Frequently mentioned forms of collaboration included agreements in primary care between general practices and community pharmacies, often established through pharmacotherapeutic audit meetings. Stakeholders also stated that HCPs with a short employment duration, such as young physicians before specialisation or temporary replacements, hindered implementation because these HCPs are often unaware of agreements and local protocols. Additionally, time constraints and high workloads were reported as barriers, limiting both adherence to protocols and adequate evaluation of prescribed therapy. Furthermore, stakeholders reported that the compatibility of the recommended action with daily routines influenced implementation. Actions such as adding prophylactic medication were easier to follow up on, as they could be integrated into CDSS. In contrast, it was reported as a barrier when recommendations required collaboration with other HCPs.

*Community pharmacist*: “*But then of course we can relatively easily discuss this with the GPs, but communication with secondary care is so much more difficult.*”

*General practitioner*: “*What I think works very well for this recommendation is that many working agreements have been established between pharmacists and GPs, in which pharmacists are mandated to add this themselves. So if the prescriber forgot, it could still be taken care of easily.*”

#### Individuals

Lastly, at the individual level, stakeholders mentioned physicians’ pharmacotherapy knowledge as a facilitator to implementation, whereas insufficient knowledge was posed as a barrier. Younger physicians were described as more aware of pharmacotherapy guidelines compared with more experienced colleagues. Geriatricians were generally well informed about contraindications. Conversely, physicians less involved in pharmacotherapy, such as surgeons or those prescribing outside their field of expertise, were considered a barrier. For detailed information on which medication safety recommendations were influenced by specific implementation factors, see Supplementary File 3.

### Barriers and facilitators for each type of recommended medication safety action

Table 2 provides a comprehensive overview of how the identified barriers and facilitators influenced the implementation of each type of recommended medication safety action. Overall, across the four most relevant CFIR domains (Fig. 1), the following key themes emerged that affected most types of actions: (1) unspecified responsibilities within the recommendation; (2) lack of information exchange between HCPs; (3) the level of collaboration between healthcare sectors; and (4) the extent to which HCPs follow protocol.

**Table 2:**
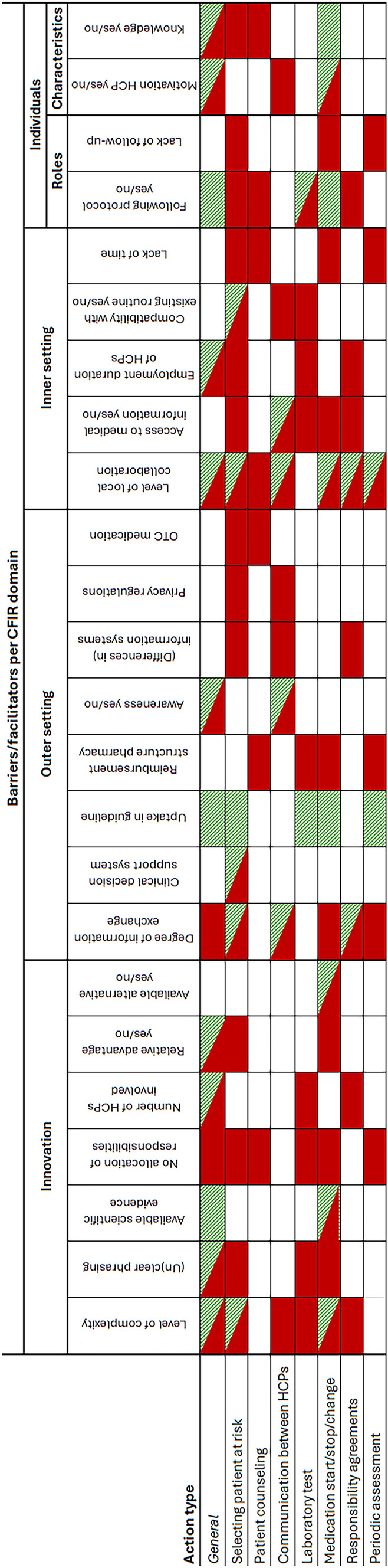
Barriers (red/solid-filled cells), facilitators (green/striped cells) or both (half-half) for each type of recommended medication safety action, categorised by CFIR domain [22]

HCP = healthcare provider; OTC = over-the-counter. General = barriers and facilitators that do not apply to a specific action within a recommendation, but apply to the recommendation in its entirety. As a selection was necessary, only factors that acted as a barrier/facilitator for four or more recommendations were included in the table. Local collaboration was defined as multidisciplinary collaboration with nearby organisations and within the region.

### Differences in barriers and facilitators between recommendations with moderate/high and low levels of implementation

Comparison of barriers and facilitators between recommendations with a moderate or high level of implementation and those with a low level of implementation revealed several differences (Table 3). Well implemented recommendations were reported to be less complex, more often supported by scientific evidence and CDSS, more frequently included in guidelines, facilitated by local collaboration, and more compatible with existing routines. In contrast, stakeholders more often mentioned poorly implemented recommendations to be hindered by unclear phrasing, a lack of available alternative medications, dependence on other HCPs, and short employment duration of HCPs.

**Table 3:** Percentages of recommendations, by level of implementation (moderate/high vs low), that were influenced by each specific implementation factor

Implementation factors per CFIR domain	Medication safety recommendations			
	High/moderate level of implementation N = 7		Low level of implementation N = 10	
	Factor functioned as a		Factor functioned as a	
	Facilitator (%)	Barrier (%)	Facilitator (%)	Barrier (%)
*Innovation*				
Available scientific evidence	71	14	10	0
Level of complexity	57	14	10	50
(Un)clear phrasing	29	14	0	50
Number of HCPs involved	29	0	0	30
Relative advantage of following recommendation yes/no	14	14	0	40
Available alternative yes/no	14	0	0	30
No allocation of responsibilities	0	29	0	40
*Outer setting*				
CDSS	57	43	0	20
Uptake in guideline	57	0	30	0
Degree of information exchange	14	57	30	50
OTC medication	0	57	0	0
Reimbursement structure pharmacy	0	14	0	40
Privacy regulations	0	29	0	20
(Differences in) information systems	0	14	0	30
Awareness yes/no	0	0	30	20
*Inner setting*				
Local collaboration	86	29	40	70
Compatibility with existing routine yes/no	43	14	0	20
Access to medical information yes/no	14	43	10	60
Lack of time	0	29	0	20
Employment duration of HCPs	0	0	10	40
*Roles of individuals*				
Following protocol yes/no	43	29	10	30
Lack of follow-up	0	29	0	20
*Characteristics of individuals*				
Motivation HCP yes/no	43	29	20	30
Knowledge yes/no	43	43	20	10

CDSS = clinical decision support systems, CFIR = Consolidated Framework for Implementation Research, HCP = healthcare provider, OTC = over-the-counter. As a selection was necessary, only factors that acted as a barrier/facilitator for four or more recommendations were included in the table. Innovation = the recommendation itself; outer setting = national influences; inner setting = influences from within the organisation and its surrounding environment; roles of individuals = how involved HCPs fulfil their roles; characteristics of individuals = characteristics of involved HCPs; implementation process = activities aimed at enhancing the implementation.

## Discussion

Five focus groups with stakeholders from diverse professional backgrounds and healthcare sectors identified a variety of barriers and facilitators affecting the implementation of medication safety recommendations. Four key themes emerged: (1) no specified responsibilities in the recommendation; (2) lack of information exchange between HCPs; (3) collaboration between HCPs across different healthcare sectors; and (4) the extent to which HCPs follow protocol. Recommendations with higher levels of implementation were reported as being less complex, more clearly phrased, more often supported by CDSS, and more often facilitated by stronger local collaboration compared to recommendations with lower implementation levels.

Several implementation factors identified in this study align with previous literature. Inadequate information exchange, particularly from secondary to primary care, has previously been reported as a major barrier [17, 18]. Compatibility with daily routines, reimbursement of care-related activities, collaboration, and CDSS have also been highlighted as important factors [19, 20, 24]. While CDSS emerged as a key facilitator, not all medication safety actions can be incorporated into these systems [25]. Certain actions require individualised assessments of multiple patient-specific factors. This was reflected by the finding that poorly implemented recommendations were complex (i.e., requiring multiple patient factors) and infrequently supported by CDSS. Even for recommendations compatible with CDSS, the range of barriers and facilitators identified suggests that CDSS alone are insufficient for successful implementation. Limitations of CDSS, such as alert fatigue and workflow disruption, should also be considered [26]. Similarly, while incorporation of recommendations into guidelines can facilitate implementation, embedding medication safety recommendations into clinical guidelines is only a first step, as numerous additional factors across multiple levels influence successful implementation [16].

The four key themes that were identified to influence implementation require further attention. First, recommendations should clearly allocate responsibilities among HCPs to facilitate coordination and collaboration across healthcare sectors. Recommendations should also include an implementation section, as evidence suggests that practical implementation instructions improve guideline uptake [27]. Such sections have become more common in recent years and should be further embraced, ideally including a plan for monitoring and evaluating implementation [28]. Second, the lack of information exchange between healthcare sectors (e.g., missing lab results, contraindications, and post-discharge medication changes) limits safe prescribing and optimal pharmaceutical care. Optimisation of electronic health record exchange and the establishment of local and national agreements on effective data exchange are needed. As a starting point, electronic information exchange between HCPs has been made mandatory in the Netherlands since 2023 [29]. Third, local collaboration across healthcare sectors should be strengthened. While some actions in moderately/well implemented medication safety recommendations can be performed by pharmacists independently, this is only possible if agreements are in place. For example, adding a proton-pump inhibitor to a non-steroidal anti-inflammatory drug without prior consultation with a physician may be permitted when agreements are in place. This underscores the importance of agreements among HCPs. Stakeholders mentioned that pharmacotherapeutic audit meetings between community pharmacists and GPs are effective. These should therefore be further utilised to establish agreements aiming to optimise medication safety. In addition, physicians should clearly define who is responsible for therapy monitoring and evaluation, especially when patients receive both primary and secondary care. Fourth, non-adherence to protocol is likely related to underlying factors such as time constraints and limited perceived benefits of protocol adherence [22]. Increasing understanding of how protocol adherence can improve practice would likely enhance compliance among HCPs.

Interestingly, many of the identified barriers and facilitators relate to the non-clinical recommendations formulated by the Harm-Wrestling workgroup and Sturkenboom et al. [4, 5]. These include encouraging collection, documentation and sharing of medical data by the government and healthcare organisations, as well as improving the layout of medical discharge letters to enhance information exchange between secondary and primary care. Although these recommendations were not formally assessed in this study, their successful implementation appears to be a prerequisite for integrating clinical medication safety actions into daily practice.

The identified barriers and facilitators in this study provide actionable insights for improving medication safety. Table 2 provides a comprehensive overview that can support HCPs and policymakers in identifying which barriers and facilitators to address. To guide this process, the ERIC-CFIR matching tool can be used to link identified barriers to implementation strategies [30]. Regarding implementation strategies, only a limited number of strategies have been applied to implement the medication safety recommendations in the Netherlands. Implementation efforts have mainly focused on integrating recommendations into CDSS, incorporation into clinical guidelines, and raising general awareness among HCPs [31]. However, insufficient efforts have been directed toward the four key themes identified in this study. Future strategies should target these key themes, thereby supporting HCPs in implementing medication safety recommendations and ultimately improving patient outcomes through safer prescribing, consistent monitoring, and clearer communication regarding responsibilities. Improved patient outcomes may not only be reflected in fewer medication-related hospitalisations but also in reduced physical and emotional burden and confusion for patients. This study focused on exploring barriers and facilitators from an implementer’s perspective, as the recommendations evaluated in this study, which are the most important medication safety recommendations in the Netherlands, are directed at healthcare professionals as the key implementers. However, it would be relevant to explore barriers and facilitators to implementing medication safety recommendations from patients’ perspectives and to explore the role that patients could play in performing medication safety actions in future research.

This study has several strengths and limitations. A strength is that it was the first to assess the implementation of medication safety recommendations across multiple healthcare sectors by including stakeholders with diverse professional backgrounds. Another strength is the evaluation of a range of medication safety recommendations, enabling a comprehensive assessment of barriers and facilitators from multiple perspectives. These strengths enhanced the applicability and relevance of the findings. However, several limitations should be acknowledged. First, the intended number of participants was not reached, and stakeholder subgroups were not equally represented. Medical specialists were underrepresented and pharmacists were overrepresented, which could have influenced the barriers and facilitators identified. However, the grouping of stakeholders across the focus groups was designed to ensure that each recommendation was discussed with the stakeholders most relevant to that specific recommendation. It should also be noted that the absence of a reported barrier and/or facilitator does not necessarily mean it has no influence on implementation. Future research could validate these findings in a larger population using different designs, such as a survey, to reduce potential selection bias. Second, data collection was not continued until data saturation was reached, as the study was part of a larger project, and the results were needed to inform subsequent methodological steps. Nevertheless, the inclusion of diverse stakeholders, sufficient discussion time, and multiple focus groups likely captured the most relevant barriers and facilitators. While additional factors may emerge in larger studies, the current findings provide a valuable foundation for understanding challenges in implementing medication safety recommendations. Finally, the study was conducted within the context of the Dutch healthcare system, which may limit generalisability. However, many recommendations are similar internationally, and several findings align with international literature suggesting broader relevance.

## Conclusion

This study explored the implementation of medication safety recommendations from the perspectives of various stakeholders, identifying a comprehensive set of barriers and facilitators. Key themes include recommendations with clearly specified responsibilities, improved exchange of patient medical data, strengthened local collaboration, and increased protocol adherence. Addressing these barriers and leveraging facilitators can enhance the implementation of medication safety recommendations, ultimately contributing to safer prescribing, better monitoring and a reduction in medication-related hospitalisations.

## Supplementary Information

Below is the link to the electronic supplementary material.

Supplementary file1 (DOCX 25 KB)

Supplementary file2 (DOCX 26 KB)

Supplementary file3 (DOCX 35 KB)

## Data Availability

The datasets generated during the current study are available from the corresponding author on reasonable request.
