# Supplementary material for: Barriers and facilitators for the implementation of medication safety recommendations: focus groups with stakeholders
Source: Int J Clin Pharm. 2026 Apr 30;48(4):1607–21. doi: 10.1007/s11096-026-02141-z (PMC13369716; doi:10.1007/s11096-026-02141-z)
Supplement: Supplementary file 1 — Supplementary file1 (DOCX 25 KB) [file 11096_2026_2141_MOESM1_ESM.docx]

**Barriers and facilitators for the implementation of medication safety recommendations: focus groups with stakeholders**

International Journal of Clinical Pharmacy

*Mirthe Oude Lansink^1,2,3^, Marcia Vervloet^4^, Lise van Tholen^5^, Marloes Dankers^6^, Mette Heringa^7^, Bart van den Bemt^1,2,3^, Liset van Dijk^4,5^, Victor Huiskes^1,2^*^,3^

1. *Department of Pharmacy, Sint Maartenskliniek, Nijmegen, the Netherlands*
2. *Department of Research, Sint Maartenskliniek, Nijmegen, the Netherlands*
3. *Department of Pharmacy, Radboudumc, Nijmegen, the Netherlands*
4. *Nivel, Netherlands Institute for Health Services Research, Utrecht, the Netherlands*
5. *Faculty of Science and Engineering, Department of PharmacoTherapy, Groningen Research Institute of Pharmacy, Epidemiology & Economics (PTEE), University of Groningen, Groningen, the Netherlands*
6. *Dutch Institute for Rational Use of Medicine, Utrecht, the Netherlands*
7. *SIR Institute for Pharmacy Practice and Policy, Leiden, the Netherlands*

Corresponding author: m.oudelansink@maartenskliniek.nl

**Supplementary File 1**

**Desktop research**

Literature

A search was conducted in PubMed and six different Dutch journals in pharmacy, general practice and nursing:

- Pharmaceutisch Weekblad
- Nederlands Platform voor Farmaceutisch Onderzoek
- Huisarts en Wetenschap
- Tijdschrift voor Praktijkondersteuners en Praktijkverpleegkundigen
- Tijdschrift voor Verzorgenden
- Verpleegkundige in praktijk en wetenschap

Search strategy

The search strategy was composed of the drugs to which the recommendations relate. For recommendations with clearly described interventions, the intervention was added to the search strategy with AND (e.g., prophylaxis, deprescribing, patient counseling). Since we aimed to assess the level of implementation in the Netherlands, the names of relevant Dutch researchers were added to the search strategy. A list of these was formulated, including researchers in general practice, geriatrics and pharmaceutical care. This list was reviewed and completed by members of the medication safety workgroup.

The structure of the search in PubMed was as followed for each recommendation: (drug [Mesh] OR drug [tiab]) AND *(intervention [Mesh] OR intervention [tiab])* AND (Dutch authors [Author]).

Since several recommendations were likely to be implemented through pharmaceutical care interventions such as clinical decision rules and medication reviews, these studies were therefore unlikely to be captured by the specific search. Therefore, an additional overarching search was performed with the following structure: (intervention [Mesh] OR intervention [tiab]) AND (Dutch authors [Author]).

Inclusion criteria

Only the articles reflecting the implementation status in the Netherlands were included. Other inclusion criteria were formulated for every recommendation separately.

Exclusion criteria

Information on the level of implementation before 2013 was not of interest, as the report by Sturkenboom et al. based their findings on data collected until 2013 (Sturkenboom MC et al., 2017). Therefore, research published before 2013 was excluded.
