# Supplementary material for: Barriers and facilitators for the implementation of medication safety recommendations: focus groups with stakeholders
Source: Int J Clin Pharm. 2026 Apr 30;48(4):1607–21. doi: 10.1007/s11096-026-02141-z (PMC13369716; doi:10.1007/s11096-026-02141-z)
Supplement: Supplementary file 2 — Supplementary file2 (DOCX 26 KB) [file 11096_2026_2141_MOESM2_ESM.docx]

**Barriers and facilitators for the implementation of medication safety recommendations: focus groups with stakeholders**

International Journal of Clinical Pharmacy

*Mirthe Oude Lansink^1,2,3^, Marcia Vervloet^4^, Lise van Tholen^5^, Marloes Dankers^6^, Mette Heringa^7^, Bart van den Bemt^1,2,3^, Liset van Dijk^4,5^, Victor Huiskes^1,2^*^,3^

1. *Department of Pharmacy, Sint Maartenskliniek, Nijmegen, the Netherlands*
2. *Department of Research, Sint Maartenskliniek, Nijmegen, the Netherlands*
3. *Department of Pharmacy, Radboudumc, Nijmegen, the Netherlands*
4. *Nivel, Netherlands Institute for Health Services Research, Utrecht, the Netherlands*
5. *Faculty of Science and Engineering, Department of PharmacoTherapy, Groningen Research Institute of Pharmacy, Epidemiology & Economics (PTEE), University of Groningen, Groningen, the Netherlands*
6. *Dutch Institute for Rational Use of Medicine, Utrecht, the Netherlands*
7. *SIR Institute for Pharmacy Practice and Policy, Leiden, the Netherlands*

Corresponding author: m.oudelansink@maartenskliniek.nl

**Supplementary File 2**

**Focus group topic guide**

Round 1: recommendations with a moderate/high level of implementation

1. Level of implementation
   - Do you agree or disagree that this recommendation is well implemented?
2. Barriers and facilitators
   1. Which factors facilitated the implementation of this recommendation?
   2. Which factors hindered the implementation of this recommendation?

Round 2: recommendations with a low level of implementation

1. Level of implementation
   - Do you agree or disagree that this recommendation is poorly implemented?
2. Barriers and facilitators
   1. Which factors facilitated the implementation of this recommendation?
   2. Which factors hindered the implementation of this recommendation?

Additional questions asked in both rounds when discussing barriers and facilitators (based on the CFIR (Damschroder et al., 2022)):

- Which factors at the level of the recommendation itself contribute to good or poor implementation of the recommendation?
  - *Think of: complexity, applicability, evidence supporting the need for the recommendation, benefits of adhering to the recommendation*
- Which external factors (outside one's own organisation) contribute to good or poor implementation of the recommendation?
  - *Think of: patient needs, network with external colleagues/institutions, policies, guidelines, reimbursement.*
- Which internal factors (within one's own organisation) contribute to good or poor implementation of the recommendation?
  - *Think of: communication within the organisation, work culture, available resources, leadership, willingness to change.*
- Which individual factors, at the level of involved healthcare professionals and other parties, contribute to good or poor implementation of the recommendation?
  - *Think of: knowledge, motivation, competencies.*
- Which process-related factors required for implementation contribute to good or poor implementation of the recommendation?
  - *Think of: planning, involvement of necessary stakeholders, reflection and evaluation.*
