# Supplementary material for: Barriers and facilitators for the implementation of medication safety recommendations: focus groups with stakeholders
Source: Int J Clin Pharm. 2026 Apr 30;48(4):1607–21. doi: 10.1007/s11096-026-02141-z (PMC13369716; doi:10.1007/s11096-026-02141-z)
Supplement: Supplementary file 3 — Supplementary file3 (DOCX 35 KB) [file 11096_2026_2141_MOESM3_ESM.docx]

**Barriers and facilitators for the implementation of medication safety recommendations: focus groups with stakeholders**

International Journal of Clinical Pharmacy

*Mirthe Oude Lansink^1,2,3^, Marcia Vervloet^4^, Lise van Tholen^5^, Marloes Dankers^6^, Mette Heringa^7^, Bart van den Bemt^1,2,3^, Liset van Dijk^4,5^, Victor Huiskes^1,2^*^,3^

1. *Department of Pharmacy, Sint Maartenskliniek, Nijmegen, the Netherlands*
2. *Department of Research, Sint Maartenskliniek, Nijmegen, the Netherlands*
3. *Department of Pharmacy, Radboudumc, Nijmegen, the Netherlands*
4. *Nivel, Netherlands Institute for Health Services Research, Utrecht, the Netherlands*
5. *Faculty of Science and Engineering, Department of PharmacoTherapy, Groningen Research Institute of Pharmacy, Epidemiology & Economics (PTEE), University of Groningen, Groningen, the Netherlands*
6. *Dutch Institute for Rational Use of Medicine, Utrecht, the Netherlands*
7. *SIR Institute for Pharmacy Practice and Policy, Leiden, the Netherlands*

Corresponding author: m.oudelansink@maartenskliniek.nl

**Supplementary File 3**

*Table 1. Barriers (red) and facilitators (green) for medication safety recommendation with a moderate to high level of implementation, stratified for recommended type of action and categorised per CFIR domain. Brackets contain more detailed information of how this barrier/facilitator influenced implementation of the recommended type of action for this specific recommendation.*

|  | **Type of action** | | | | | | | |
| --- | --- | --- | --- | --- | --- | --- | --- | --- |
| **Medication safety recommendations** | **General** | **Selecting patient at risk** | **Patient counselling** | **Communication between HCP’s** | **Laboratory test** | **Medication start/stop/change** | **Responsibility agreements** | **Periodic assessment** |
| Pharmacy informs anticoagulant service about initiation and discontinuation of medication interacting with coumarin | **Recommendation**  Available scientific evidence Number of HCPs involved  Level of complexity  Relative advantage yes/no  **External factors**  Initial conditions  Government pressure Education  Policy  **Roles**  Champions in the field  **Characteristics**  Motivation (HCP) | **External factors**  OTC medication  **Internal factors**  Compatibility with existing routine (pharmacy) |  | **External factors**  Degree of information exchange  **Internal factors**  Access to medical information yes/no  Level of local collaboration  Compatibility with existing routine |  |  |  |  |
| Adding PPI to patients using NSAID/acetyl salicylic acid at risk of gastrointestinal bleeding. Patients are informed about alarm symptoms | **Recommendation**  Available scientific evidence  **External factors**  Uptake in guideline  Benchmarking (reimbursement, government)  Education  **Internal factors**  Level of local collaboration  **Characteristics**  Knowledge (GPs)  Motivation (HCPs general)  Motivation (GP) | **Recommendation**  Level of complexity (amount of patient information required)  **External factors**  CDSS  OTC medication  **Internal factors**  Supporting materials  Lack of time  **Roles**  Patient’s use of someone else’s medication  Following protocol yes/no (GP) | **External factors**  OTC medication  **Internal factors**  Supporting materials  **Characteristics**  Capability (pharmacy technician)  Knowledge (patient) |  |  | **Recommendation**  NSAID side-effects  PPI side-effects  **External factors**  Drug reimbursement  Reimbursement structure pharmacy  Drug reimbursement  **Internal factors**  Collaboration (GP-pharmacy)  Medication reviews  **Characteristics**  Capability (pharmacy technicians) |  |  |
| Informing patient about the importance of therapy adherence to PPI. This PPI is discontinued along with the NSAID/acetyl salicylic acid | **Internal factors**  Level of local collaboration | **External factors**  CDSS  (pharmacy)  Degree of information exchange (second to first line care)  OTC medication  **Internal factors**  Access to medical information yes/no  **Roles**  Lack of follow-up | **Roles**  Following protocol yes/no (hospital discharge, pharmacy technician)  **Characteristics**  Counselling skills (pharmacy technician)  Knowledge (patient) |  |  | **Recommendation**  Available scientific evidence  Available scientific evidence  **External factors**  Drug reimbursement  **Internal factors**  Medication reviews  **Characteristics**  Motivation (patient) |  |  |
| No NSAID in case of contraindications. If there is no alternative, NSAID is prescribed for short-term use with kidney function monitoring. Patients are informed about alarm symptoms | **Recommendation**  (Un)clear phrasing  (Un)clear phrasing  **Internal factors**  Level of local collaboration  **Roles**  Alert HCP  **Characteristics**  Knowledge HCP  Knowledge (medical specialists) | **Recommendation**  Relative advantage yes/no  **External factors**  Degree of information exchange (lab)  CDSS  CDSS (frequency, timing, contra-indication)  OTC medication  Degree of information exchange  Privacy regulations  **Internal factors**  Level of local collaborations  Access to medical information yes/no (GP, pharmacy) | **Recommendation**  No allocation of responsibilities among HCPs  **Characteristics**  Capability (pharmacy technicians) |  | **External factors**  Organization lab tests (timing, pharmacist authorities) | **Recommendation**  No allocation of responsibilities  **External factors**  Degree of information exchange (second to first line care)  **Internal factors**  Level of local collaboration  Level of local collaboration  Access to medical information yes/no (pharmacist, GP)  **Roles**  Assumptions regarding other HCPs tasks  **Characteristics**  Motivation (GP) |  |  |
| Osteoporosis prophylaxes is initiated when indicated due to corticosteroid use. This prophylaxis is discontinued along with the corticosteroid if no longer indicated | **Recommendation**  Number of HCPs involved  Level of complexity  Available scientific evidence  **External factors**  Uptake in guideline  **Roles**  Following protocol yes/no | **External factors**  (Difference in) information systems  CDSS (timing)  **Internal factors**  Compatibility with existing routine  Level of local collaboration  Access to medical information yes/no (pharmacy) |  |  |  | **Recommendation**  Level of complexity  Level of complexity (frequent short-term use)  **External factors**  Drug reimbursement  Drug reimbursement |  |  |
| Adding laxatives to opioid therapy, and evaluation whether the current laxative is sufficient to prevent obstipation | **Recommendation**  Clear phrasing  Level of complexity  Available scientific evidence  **External factors**  Benchmarking (reimbursement)  Uptake in guideline  Education  **Roles**  Alert (pharmacy)  **Characteristics**  Motivation (HCPs general) | **Recommendation**  Level of complexity  **Internal factors**  Compatibility with existing routine (pharmacy)  **External factors**  CDSS  CDSS (frequency)  Degree of information exchange (second to first line care) |  |  |  | **Recommendation**  Level of complexity  **External factors**  Benchmarking (reimbursement)  Pharmacist authorities  **Internal factors**  Level of local collaboration  Lack of time  **Roles**  Following protocol yes/no (GP)  **Characteristics**  Motivation (GP)  Knowledge (GP)  Motivation (patient) |  | **Recommendation**  No allocation of responsibilities  **Roles**  Lack of follow-up |
| Glibenclamide is not to be prescribed to patients ≥75 years old due to hypoglycaemic risk |  |  |  |  |  | **Recommendation**  Available alternative yes/no  **External factors**  Uptake in guideline  **Roles**  Following protocol yes/no |  |  |

*CDSS= clinical decision support system; GP= general practitioner; HCP= healthcare provide; NSAID= non-steroidal anti-inflammatory drug; OTC= over-the-counter. PPI= proton pump inhibitor.*

*Table 2. Barriers (red) and facilitators (green) for medication safety recommendation with a low level of implementation, stratified for recommended type of action and categorised per CFIR domain. Brackets contain more detailed information of how this barrier/facilitator influenced implementation of the recommended type of action for this specific recommendation.*

|  | **Type of action** | | | | | | | |
| --- | --- | --- | --- | --- | --- | --- | --- | --- |
| **Medication safety recommendation** | **General** | **Selecting patient at risk** | **Patient counselling** | **Communication between HCP’s** | **Laboratory test** | **Medication start/stop/change** | **Responsibility agreements** | **Periodic assessment** |
| Psychotropic drugs are only initiated in elderly when there is a strict indication. Fall risk in elderly is assessed by the GP. | **Recommendation**  Level of complexity  Relative advantage yes/no  Unclear phrasing  No allocation of responsibilities  **External factors**  Awareness yes/no | **Recommendation**  Level of complexity (individual assessment)  **External factors**  Uptake in guideline  CDSS  **Internal factors**  Access to medical information yes/no (pharmacy)  **Roles**  Alert (GP) |  |  |  | **Recommendation**  Unclear phrasing  Available alternative yes/no  **Roles**  Alert (GP) |  | **External factors**  Uptake in guideline  **Internal factors**  Training (GPs)  **Roles**  Alert (GP) |
| Psychotropic drugs are not continued unnecessarily. The prescriber evaluates this periodically after initiation. Psychotropic and cardiovascular drugs are evaluated yearly in case of long-term use | **Recommendation**  Number of HCPs involved  **External factors**  Awareness yes/no  Policy  **Internal factors**  Level of local collaboration | **Recommendation**  Unclear phrasing  **External factors**  Privacy regulations  **Internal factors**  Compatibility with working routine  Employment duration of HCPs (GP)  **Roles**  Following protocol yes/no (GP)  Timing of acting  Domain thinking (medical specialists)  Information from patient |  |  |  | **Recommendation**  Level of complexity (individual assessment)  Available alternative yes/no  **Outer setting**  Reimbursement structure pharmacy  **Internal factors**  Lack of time (GP, pharmacy)  Level of local collaboration |  | **External factors**  Reimbursement structure pharmacy  Lack of time (GP, medical specialist, pharmacy)  Degree of information exchange (second to first line care)  **Internal factors**  Collaboration  (responsibility agreements)  **Roles**  Lack of follow-up  Information from patient |
| Chronic users of benzodiazepines or related substances are encouraged to discontinue or reduce the dosage by terms of a discontinuation letter | **External factors**  Awareness yes/no  Uptake in guideline  **Internal factors**  Level of local collaboration (responsibility agreements)  **Characteristics**  Knowledge (GPs)  **Process**  Informing colleagues |  | **Internal factors**  Lack of time (GP) |  |  | **Recommendation**  Available alternative yes/no  Relative advantage yes/no  **Characteristics**  Motivation (patient) |  |  |
| Informing patients using an anticoagulant about the risks of intercurrent diseases or changes in lifestyle/nutrition, and alarm symptoms of a gastrointestinal bleeding | **Recommendation**  Unclear phrasing | **Recommendation**  No allocation of responsibilities  **Internal factors**  Level of local collaboration  Level of local collaboration  **Roles**  Information by HCP (repetition)  Alert  Information by HCP (amount) |  |  |  |  |  |  |
| Monitoring of sodium/potassium levels and kidney function after initiation of a diuretic or a renin-angiotensin system inhibitor in patients at risk for electrolyte disorder or decreased kidney function | **Recommendation**  Relative advantage yes/no  Level of complexity  **External factors**  Degree of information exchange (second to first line care; lab)  **Internal factors**  Employment duration of HCPs (nurses)  Created workflow  Level of local collaboration  Employment duration of HCPs (medical residents)  **Characteristics**  Motivation | **Recommendation**  Level of complexity  **External factors**  Degree of information exchange (lab)  Degree of information exchange (second first line care; lab)  CDSS  **Internal factors**  Created workflow  Access to medical information yes/no (pharmacy) | **Internal factors**  Supporting materials | **External factors**  Privacy regulations  Collaboration | **Recommendation**  No allocation of responsibilities  Number of HCPs needed  Unclear phrasing  Level of complexity (timing)  **External factors**  Uptake in guideline  Pharmacist authorities  Collaboration (responsibility agreements)  Reimbursement structure pharmacy  Policy  **Internal factors**  Compatibility with existing routine  Access to medical information yes/no (pharmacy)  **Roles**  Following protocol yes/no  Following protocol yes/no  Information by pharmacy technician (structure)  **Process**  Initiatives (local)  Initiatives (national) |  |  |  |
| Informing patients at risk for an electrolyte disorder about alarm symptoms and risk situations (e.g., infection, diarrhoea | **Recommendation**  Unclear phrasing | **Recommendation**  No allocation of responsibilities  **Internal factors**  Level of local collaboration  Level of local collaboration  **Roles**  Information by HCP (repetition)  Alert  Information by HCP (amount)  **Characteristics**  Knowledge (GP, pharmacy) | **Roles**  Information by pharmacy technician (structure) |  |  |  |  |  |
| Monitoring patients with risks at electrolyte disorders, kidney insufficiency, syncope or hypotension, especially in case of nausea. Laboratory tests must be collected, documented and shared with involved HCPs |  | **External factors**  Degree of information exchange  **Internal factors**  Access to medical information yes/no (pharmacy)  **Roles**  Information from patient |  | **External factors**  (Difference in) information systems  Degree of information exchange | **External factors**  Organization lab tests  **Internal factors**  Access to medical information yes/no (pharmacy)  Employment duration of HCPs (GPs) |  |  |  |
| Users of oral blood glucose-lowering sulfonylurea derivates are informed about the risks of unusual physical exertion and nutrition changes, and how to manage these factors. Patients are informed about alarm symptoms in case of hypoglycaemic risks | **Recommendation**  Relative advantage |  | **External factors**  Reimbursement structure pharmacy  **Internal factors**  Level of local collaboration  Supporting materials  **Characteristics**  Knowledge (younger GPs) |  |  |  |  |  |
| Antithrombotic medication, and combinations of, are prescribed only on strict indication.  The indication and therapy duration of antithrombotic and other risk medication are communicated by the prescriber to other involved HCPs | **Recommendation**  Available scientific evidence  **External factors**  Awareness yes/no (pharmacy)  **Characteristics**  Motivation (pharmacists) | **External factors**  Privacy regulations  **Internal factors**  Existing workflow |  | **Recommendation**  Level of complexity  **External factors**  Degree of information exchange  Awareness yes/no  Degree of information exchange  Awareness yes/no  Privacy regulations  Degree of information exchange (second to first line care)  Collaboration  **Internal factors**  Access to medical information yes/no (hospital)  Access to medical information yes/no  **Characteristics**  Motivation (GPs, medical specialists) |  | **Recommendation**  Available scientific evidence  **Roles**  Lack of follow-up (GP, medical specialist) |  |  |
| Prescribers establish who holds primary responsibility for a patient’s therapy, document this, and communicate this to involved HCPs, including the pharmacist. When initiating high-risk medication not intended for long-term use, the patient, other prescribers and the pharmacist are informed about the intended therapy duration. The responsibility for periodic monitoring and evaluation of medication initiated in secondary care is discussed and documented. Elderly with polypharmacy receive periodic medication reviews | **Recommendation**  Unclear phrasing  **Characteristics**  Motivation (GP) |  |  | **External factors**  Degree of information exchange (general; second to first line care)  (Difference in) information systems  **Internal factors**  Level of local collaboration  Level of local collaboration |  |  | **Recommendation**  Number of HCPs involved  Level of complexity (multimorbidity, individual assessment needed)  **External factors**  Degree of information exchange  Degree of information exchange (general; second to first line care)  (Difference in) information systems  **Internal factors**  Level of local collaboration  Created workflow  Level of local collaboration  Employment duration of HCPs  Access to medical information yes/no (GP)  **Roles**  Assumptions regarding other HCP’s tasks  Following protocol yes/no (medical specialists) | **External factors**  Reimbursement structure Pharmacy  **Internal factors**  Level of local collaboration |

*CDSS= clinical decision support system; GP= general practitioner; HCP= healthcare provider.*
